# The prevalence of pulmonary airway lesions is high in HTLV-1-positive rheumatoid arthritis: a cross-sectional observational study

**DOI:** 10.1093/rap/rkag046

**Published:** 2026-04-07

**Authors:** Chihiro Iwao, Kunihiko Umekita, Masatoshi Kimura, Masayuki Murai, Kosho Iwao, Risa Kudou, Takeshi Kawaguchi, Yayoi Hashiba, Michikazu Nakai, Toshihiko Hidaka, Taiga Miyazaki

**Affiliations:** Division of Respirology, Rheumatology, Infectious Diseases, and Neurology, Department of Internal Medicine, University of Miyazaki, Miyazaki, Japan; Division of Respirology, Rheumatology, Infectious Diseases, and Neurology, Department of Internal Medicine, University of Miyazaki, Miyazaki, Japan; Division of Respirology, Rheumatology, Infectious Diseases, and Neurology, Department of Internal Medicine, University of Miyazaki, Miyazaki, Japan; Division of Respirology, Rheumatology, Infectious Diseases, and Neurology, Department of Internal Medicine, University of Miyazaki, Miyazaki, Japan; Division of Respirology, Rheumatology, Infectious Diseases, and Neurology, Department of Internal Medicine, University of Miyazaki, Miyazaki, Japan; Division of Respirology, Rheumatology, Infectious Diseases, and Neurology, Department of Internal Medicine, University of Miyazaki, Miyazaki, Japan; Division of Respirology, Rheumatology, Infectious Diseases, and Neurology, Department of Internal Medicine, University of Miyazaki, Miyazaki, Japan; Institute of Rheumatology, Miyazaki Zenjinkai Hospital, Miyazaki, Japan; Department of Statistics and Data Management, Faculty of Medicine, University of Miyazaki, Miyazaki, Japan; Institute of Rheumatology, Miyazaki Zenjinkai Hospital, Miyazaki, Japan; Division of Respirology, Rheumatology, Infectious Diseases, and Neurology, Department of Internal Medicine, University of Miyazaki, Miyazaki, Japan

**Keywords:** human T cell leukaemia virus type 1, rheumatoid arthritis, pulmonary airway lesions, high-resolution computed tomography, bronchiolitis

## Abstract

**Objective:**

To investigate whether human T cell leukaemia virus type 1 (HTLV-1) infection increases the risk of respiratory complications in patients with RA, focusing on high-resolution computed tomography (HRCT) findings and clinical features.

**Methods:**

We retrospectively analysed 30 HTLV-1-positive patients with RA enrolled in the HTLV-1 RA Miyazaki Registry who had undergone chest HRCT. For each patient, three age- and sex-matched HTLV-1-negative RA patients (*n* = 90) were recruited as controls. We compared pulmonary abnormalities on HRCT, RA disease activity, medication use and systemic complications between the groups. In the HTLV-1-positive group, we also assessed the relationship between HTLV-1 proviral load (PVL) and pulmonary findings.

**Results:**

Although age and disease duration were similar between groups at the time of HRCT, HTLV-1-positive patients had significantly higher disease activity (CDAI: 6.4 *vs* 3.3, *P* = 0.01; SDAI: 7.28 *vs* 3.46, *P* = 0.01), greater glucocorticoid use (70% *vs* 36%) and lower biologics use (33% *vs* 61%). Abnormal pulmonary findings were observed in ≈50% of both groups; however, bronchiolitis was more frequent in HTLV-1-positive patients, while interstitial lung disease patterns were comparable. HTLV-1 PVL did not differ significantly based on the presence or absence of pulmonary lesions.

**Conclusion:**

HTLV-1 infection may link to an increased prevalence of airway lesions, such as bronchiolitis and bronchiectasis, in RA patients. These findings suggest that screening for HTLV-1 should be considered in RA patients, especially those with respiratory symptoms or complications.

**Key messages** 

- HTLV-1 infection in RA patients may increase the prevalence of airway lesions like bronchiolitis and bronchiectasis.
- HTLV-1-positive RA patients show higher disease activity and steroid use despite similar interstitial lung disease prevalence.
- Screening for HTLV-1 infection in patients with RA may help identify those at risk of airway complications.

## Introduction

RA, a systemic autoimmune disease, is characterized by chronic inflammatory arthritis and progressive bone destruction, as well as extra-articular manifestations, lung involvement being the most common [1]. Pulmonary complications in RA include a wide spectrum of conditions, ranging from interstitial lung disease (ILD), pleuritis and airway inflammation to pulmonary nodules [2], with substantial epidemiological evidence regarding the life-threatening nature of these respiratory complications [3]. Furthermore, pulmonary involvement in RA is a known risk factor for the development of respiratory infections, such as *Pneumocystis jirovecii* pneumonia and pneumococcal pneumonia. Importantly, the presence of pulmonary complications is considered a poor prognostic factor in RA [3]. Current European Respiratory Society (ERS)/EULAR guidelines for CTD-associated ILD do not recommend routine high-resolution CT (HRCT) screening before starting antirheumatic therapy, and HRCT is not required for all patients [4]. However, patients who may have lung involvement should undergo specific respiratory assessments, such as imaging, when it is medically necessary. To date, pulmonary manifestations in RA remain heterogeneous and the factors predisposing to airway-dominant involvement remain unclear.

Human T cell leukaemia virus type 1 (HTLV-1) is a retrovirus that causes adult T cell leukaemia/lymphoma (ATL), HTLV-1-associated myelopathy/tropical spastic paraparesis (HAM/TSP) and HTLV-1-associated uveitis (HAU) [5]. In addition, HTLV-1 infection is associated with other conditions such as seborrheic dermatitis, Sjögren’s syndrome, HTLV-1-associated arthropathy, fibromyalgia and cervical cancer [6]. In the respiratory system, HTLV-1-associated bronchoalveolitis (HABA) is recognized as a form of HTLV-1-related pulmonary involvement [7, 8]; however, the pathophysiology of HABA remains unclear, with very limited data regarding its incidence and clinical characteristics in HTLV-1 carriers. HTLV-1 establishes a lifelong persistent infection in CD4-positive T lymphocytes and differs virologically and immunologically from acute respiratory viruses, such as respiratory syncytial virus, and from EBV, which primarily establishes latent infections in B cells. Therefore, the current study focused on analysing effects specific to HTLV-1.

Although both RA and HTLV-1 infection can independently cause pulmonary lesions, it remains unclear whether lung involvement is more frequent and more severe in patients with RA who are living with HTLV-1. To date, no study has investigated the prevalence and nature of respiratory complications in HTLV-1-positive patients with RA. Accordingly, this study aimed to evaluate the characteristics of pulmonary lesions in HTLV-1-positive patients with RA compared with HTLV-1-negative patients with RA using HRCT.

## Methods

### Study setting and centres

This study was conducted in Miyazaki Prefecture, Japan, an area endemic for HTLV-1. Miyazaki University Hospital is a tertiary referral centre with specialized expertise in rheumatology, respiratory medicine and infectious diseases. The Institute of Rheumatology at Miyazaki Zenjinkai Hospital is a high-volume specialized centre that provides comprehensive care for patients with RA. Together, these institutions deliver continuous care to ≈1500 patients with RA, enabling the identification and longitudinal follow-up of HTLV-1-positive patients with RA.

### Type of RA patients

The registry includes adult patients with RA diagnosed according to the 1987 American College of Rheumatology (ACR) classification criteria [9, 10] who were receiving standard antirheumatic treatment, including methotrexate and/or biologic DMARDs (bDMARDs), in accordance with established treatment guidelines [11–13]. Patients were managed routinely and underwent regular follow-up at the participating centres.

### Registry design

The HTLV-1 RA Miyazaki Registry was established in December 2017 at Miyazaki University Hospital and Miyazaki Zenjinkai Hospital as an extension of the previously reported HTLV-1 RA Miyazaki Cohort Study [14]. The registry is an observational clinical registry designed to address two primary research questions: the impact of HTLV-1 infection on the clinical characteristics of patients with RA and whether antirheumatic therapies, including immunosuppressive agents, influence the risk of developing ATL in HTLV-1-positive patients with RA.

A total of 858 patients with RA were enrolled in the registry and screened for HTLV-1 infection. Among them, 85 HTLV-1-positive patients with RA were identified and enrolled by April 2022. Written informed consent was obtained from all participants, and patients were followed longitudinally with periodic clinical assessments and data collection at Miyazaki University Hospital and Miyazaki Zenjinkai Hospital.

For the present study, HTLV-1-positive patients with RA were selected from the registry if they had undergone HRCT and had periodic measurements of HTLV-1 proviral load (PVL) using quantitative PCR. Patients were excluded if HRCT had previously been performed to diagnose or evaluate pulmonary infections, such as pneumonia or tuberculosis, or if the current HRCT was conducted during cancer treatment to assess metastatic or primary lung cancer lesions. Accordingly, 30 of the 85 HTLV-1-positive patients were enrolled in this study. Additionally, three age- and sex-matched HTLV-1-negative patients with RA who had satisfied the inclusion criteria from 773 HTLV-1-negative RA patients were selected as controls for each HTLV-1-positive patient; age was matched to within 5 years. To avoid recruitment and sampling bias, control participants were randomly selected from 773 HTLV-1-negative patients with RA based on sex and age and no other clinical information was used. Finally, 90 HTLV-1-negative patients with RA were enrolled as controls in the present study. All clinical information, such as RA disease activity and antirheumatic regimen, was collected from the participant’s medical records.

The study followed the Ethical Guidelines for Medical and Health Research Involving Human Subjects [15] and the protocol was approved by the Research Ethics Committees of the University of Miyazaki Hospital (approval O-0236) and Miyazaki Zenjinkai Hospital.

### Clinical assessment of RA

In this study, clinical data related to RA were periodically recorded in the registry database during the observation period from 1 March 2018 to 6 March 2019. From these records, the most recent clinical findings and laboratory values within 3 months before or after the HRCT were extracted. According to the EULAR guidelines, the 28-joint Disease Activity Score using the ESR (DAS28-ESR), the Simple Disease Activity Index (SDAI) and the Clinical Disease Activity Index (CDAI) were used to evaluate RA disease activity [16, 17], a visual analogue scale (VAS) was used to evaluate pain (pain-VAS) and the patient global assessment (PtGA) and physician global assessment (PhGA) were used to evaluate RA disease activity [18]. Remission (REM), low disease activity (LDA), moderate disease activity (MDA) and high disease activity (HDA) were defined as follows according to established criteria: REM <2.6, LDA 2.6–<3.2, MDA 3.2–≤5.1 and HDA >5.1 according to the DAS28-ESR; REM ≤2.8, LDA >2.8–≤10, MDA >10–≤22 and HDA >22 according to the CDAI; and REM ≤3.3, LDA >3.3–≤11, MDA >11–≤26 and HDA >26 according to the SDAI [19]. Physical function was assessed using the HAQ Disability Index (HAQ-DI).

### Assessment of chest findings on HRCT

HRCT examinations were performed with either a Aquilion One (Canon Medical Systems, Ōtawara, Japan), an IQon Spectral CT (Philips Healthcare, Andover, MA, USA) or a Blight Speed Elite Pro (GE Medical Systems, Chicago, IL, USA) unit. Thin-section CT was performed with 1–2.5 mm collimation at 10-mm intervals. For analysis of the lung parenchyma, a lung window was applied (window level −550 HU; window width 1800 HU). For interpretation of the mediastinum, images were set for a mediastinal window (window level 45 HU; isolated window width 350 HU). HRCT findings were evaluated based on radiology reports generated by board-certified radiologists during routine clinical practice in accordance with the Fleischner Society criteria. Furthermore, all HRCT images were independently reviewed by a clinician specializing in both pulmonology and rheumatology who assessed all pulmonary imaging parameters and collected imaging findings using standardized definitions. Imaging findings and classifications were subsequently reviewed and confirmed by a board-certified radiologist and any discrepancies were resolved through discussion to reach a final consensus. Radiological findings assessed included bronchiolitis, bronchiectasis, reticular pattern, interlobular septal thickening, ground-glass attenuation, honeycombing, pleural thickening, lung nodules and emphysematous bullae. Bronchiolitis was defined on HRCT as small airway abnormalities, including centrilobular nodules and peribronchiolar opacities. Bronchiectasis was defined as irreversible bronchial dilatation with an internal diameter greater than that of the accompanying pulmonary artery, in accordance with the Fleischner Society criteria. Bronchiectasis was further classified as traction or freestanding based on its association with ILD. Our analysis included only freestanding (non-traction) bronchiectasis and excluded traction bronchiectasis. Any remaining discrepancies between the radiology reports and the independent assessments were resolved through discussions, with the final classification being determined via consensus.

### Measurement of HTLV-1 PVL

This study utilized HTLV-1 PVL values measured during the 3 months preceding and following the date of HRCT imaging. The HTLV-1 PVL value was measured based on the peripheral blood monocytes (PBMCs) separated by Ficoll-based density gradient centrifugation. DNA was purified from PBMCs and real-time PCR was performed to measure the HTLV-1 *pX* region and the human albumin gene using the Light Cycler 2.0 thermal cycler (Roche Diagnostics, Mannheim, Germany) [20]. HTLV-1 PVL values in PBMCs were measured in duplicates.

### Statistical analyses

Statistical analyses were performed using GraphPad Prism version 6.0 (GraphPad Software, Boston, MA, USA) and Stata version 19 (StataCorp, College Station, TX, USA). Participant characteristics were summarized as the mean (s.d.) or the median with interquartile range (IQR) for continuous variables, depending on data distribution, and as frequencies and percentages for categorical variables. Normality of continuous variables was assessed using the Shapiro–Wilk test. Non-normally distributed continuous variables were analysed using the Mann–Whitney U test, whereas normally distributed continuous variables were analysed using the Student’s *t*-test. Categorical variables were analysed using Fisher’s exact test. Propensity score matching was performed using a 1:1 nearest-neighbour matching method without replacement based on a logit model, with a calliper width of 0.001. The standard mean difference (SMD) was then calculated, with values <0.10 indicating good balance. Adjusted variables included sex, age and bDMARD use. *P*-values <0.05 were considered statistically significant.

## Results

### Clinical characteristics of study patients at the time of HRCT

Table 1 shows the clinical characteristics of the study patients at the time of HRCT. The disease activity of all patients was well controlled by treatment with DMARDs (including MTX and/or biologic agents) as recommended by the ACR/EULAR guidelines [12, 13]. There were no between-group differences in terms of ACPA or RF seroprevalence and CRP and ESR values. There were fewer cases of tender joint count and swollen joint count in most of the participants. According to the EULAR criteria, the CDAI, SDAI and DAS28-ESR values in both groups were within the REM or LDA range, and none of the patients met the criteria for MDA or HDA. However, DASs were higher in the HTLV-1-positive group than in the HTLV-1-negative controls, with statistically significant differences observed for CDAI and SDAI (DAS28-ESR: 3.00 *vs* 2.56, *P* = 0.06; CDAI: 6.40 *vs* 3.30, *P* = 0.01; SDAI: 7.28 *vs* 3.47, *P* = 0.01). Likewise, the pain-VAS and PtGA values were higher for the HTLV-1-positive patients than for the HTLV-1-negative controls (pain-VAS: 27 *vs* 12, *P* = 0.01; PtGA: 30 *vs* 14, *P* = 0.03). Although the median HAQ-DI score tended to be higher in the HTLV-1-positive group, the difference was not statistically significant (0.88 *vs* 0.38, *P* = 0.28).

**Table 1:** Baseline characteristics of HTLV-1-negative and -positive patients with RA.

Characteristics	HTLV-1-negative RA (*n* = 90)	HTLV-1-positive RA (*n* = 30)	*P*-value^h^
Female, *n* (%)	75 (83)	24 (80)	0.78
Age, mean (s.d.), years	73 (8.55)	70 (9.28)	0.04
Age at RA diagnosis, mean (s.d.), years	55 (13.26)	56 (11.55)	0.82
RA duration, median (IQR), years	16 (9.00–25.00)	14 (7.00–20.00)	0.11
Late-onset RA, *n* (%)	35 (38)	10 (33)	0.67
Smoker, *n* (%)	16/83 (19)	8 (27)	0.44
RF positive, *n* (%)^a^	60 (67)	21 (70)	0.82
ACPA positive, *n* (%)^b^	74/88 (84)	23 (77)	0.41
ACPA level, median (IQR), U/ml	61 (7.00–101.00)	29 (5.40–101.00)	0.29
RA stage, *n*	I: 1; II: 16; III: 23; IV: 50	I: 2; II: 9; III: 7; IV: 12	0.13
RA functional class, *n*	I: 52; II: 22; III: 9; IV: 7	I: 13; II: 14; III: 3; IV: 0	0.07
RA disease activity			
MMP-3, median (IQR)	73 (47.20–134.00)	106 (47.70–209.50)	0.27
CRP, median (IQR), mg/dl	0.11 (0.04–0.33)	0.14 (0.05–0.82)	0.37
DAS28-ESR, mean (s.d.)	2.56 (1.05)	3.00 (1.08)	0.06
CDAI, median (IQR)	3.30 (1.15–5.85)	6.40 (3.05–9.85)	0.01
SDAI, median (IQR)	3.46 (1.50–6.38)	7.28 (3.22–11.30)	0.01
Pain, 100-mm VAS, median (IQR)	12.00 (4.00–30.00)	27.00 (10.00–51.00)	0.02
PGA, 100-mm VAS, median (IQR)	14.00 (5.00–31.00)	30.00 (13.00–53.00)	0.03
EGA, 100-mm VAS, median (IQR)	5.00 (3.00–11.00)	10.00 (3.00–15.00)	0.05
HAQ-DI, median (IQR)	0.38 (0.00–1.40)	0.88 (0.12–1.50)	0.28
Treatment, *n* (%)			
Corticosteroid	32 (36)	21 (70)	<0.01
Methotrexate	47 (52)	10 (33)	0.07
Biologics	55 (61)	10 (33)	<0.01
Tacrolimus	19 (21)	10 (33)	0.22
Comorbidities, *n* (%)			
CTD	22 (24)	8 (27)	0.81
Interstitial pneumonia	26 (29)	10 (33)	0.65
Uveitis	2 (2)	1 (3)	>0.99
Skin disease	1 (1)	0	>0.99
HAM	–	1 (3)	
ATL	–	1 (3)	
Diabetes	16 (18)	7 (23)	0.59
Malignancy	15 (17)	6 (20)	0.78

a RF positivity (>15 IU/ml).

b ACPA positivity (≥4.5 U/ml).

c *n* = 27.

d *n* = 88.

e *n* = 28.

f *n *= 29.

g *n* = 87.

h Significance level of the Fisher’s exact test for categorical variables and the Mann–Whitney U test for continuous variables.

The proportion of patients receiving corticosteroids was higher in the HTLV-1-positive group than in HTLV-1-negative group (70% *vs* 36%, *P* < 0.01), whereas the proportion of patients receiving MTX and biologic agents tended to be lower in the HTLV-1-positive group (33% *vs* 52%, *P* = 0.07 and 33% *vs* 61%, *P* < 0.01, respectively). There were no statistically significant differences between the two groups regarding the prevalence of comorbidities such as CTDs, diabetes mellitus, skin diseases, uveitis and malignancies. To assess the robustness of our findings, additional propensity score matching (PSM) analyses were performed. All SMD values for the adjusted variables were <0.10, indicating adequate covariate balance after PSM. One analysis adjusted for sex, age and bDMARD use (PSM1), while another further adjusted for disease activity (DAS28-ESR, CDAI and SDAI; PSM2). In both matched analyses, the prevalence of bronchiolitis remained higher in the HTLV-1-positive group than in HTLV-1-negative group. These results are presented in Supplementary Tables PSM1 and PSM2.

### Reasons for performing HRCT

Table 2 summarizes the reasons for performing HRCT in both groups. Follow-up of abnormal HRCT findings and routine screening for respiratory complications after RA diagnosis were the predominant indications for HRCT. Three patients underwent HRCT for follow-up of previous malignancies, including two with malignant lymphoma and one with renal cancer. The distribution of indications for HRCT did not differ substantially between the HTLV-1-positive and HTLV-1-negative groups.

**Table 2:** Reasons for having an HRCT scan.

Reasons	HTLV-1-negative RA (*n* = 90), *n* (%)	HTLV-1-positive RA (*n* = 30), *n* (%)	*P*-value
Follow-up of lung lesions	37 (41)	10 (33)	0.52
Routine checkup	41 (46)	9 (30)	0.20
Follow-up of cancer	2 (2)	4 (13)	0.03
Screening before starting biologics	5 (6)	4 (13)	0.23
Abnormal findings with plain X-ray	4 (4)	3 (10)	0.36
Unknown	1 (1)	0	>0.99

Statistical analysis was performed using Fisher’s exact test.

### Abnormal findings in pulmonary HRCT in HTLV-1-positive and HTLV-1-negative patients with RA

Several abnormal pulmonary HRCT findings, excluding ILD, were observed in both groups (Table 3). There was no significant difference in the overall prevalence of abnormal pulmonary findings between HTLV-1-positive and HTLV-1-negative patients (47% *vs* 53%, *P* = 0.53). However, the prevalence of bronchiolitis was significantly higher in HTLV-1-positive patients than in HTLV-1-negative patients (37% *vs* 13%, *P* < 0.01). Findings related to bronchiectasis were numerically more frequent in the HTLV-1-positive group, although this difference did not reach statistical significance (27% *vs* 12%, *P* = 0.09). ILD patterns, including usual interstitial pneumonia (UIP), non-specific interstitial pneumonia (NSIP) and organizing pneumonia, were observed in ≈30% of patients in both groups, with no significant between-group differences.

**Table 3:** Pulmonary HRCT findings in HTLV-1-positive/negative patients with RA.

Findings	HTLV-1-negative RA (*n* = 90), *n* (%)	HTLV-1-positive RA (*n* = 30), *n* (%)	*P*-value
Abnormal findings (total)	48 (53)	14 (47)	0.53
Bronchiolitis	12 (13)	11 (37)	<0.01
Bronchiectasis	12 (13)	9 (27)	0.09
Reticular pattern	21 (23)	7 (23)	1.00
Interlobular septum thickening	19 (21)	7 (23)	0.80
Ground glass attenuation	8 (9)	4 (13)	0.48
Honeycombing	12 (13)	4 (13)	0.87
Pleural thickening	14 (12)	3 (10)	0.74
Lung nodules	5 (6)	2 (7)	0.82
Emphysematous bullae	4 (4)	1 (3)	0.79
ILD CT pattern	*n* = 26	*n* = 10	
UIP	11 (42)	4 (40)	0.65
NSIP	4 (15)	1 (10)	0.79
Organizing pneumonia	0	1 (10)	0.08
Mild, unclassifiable	11 (42)	4 (40)	0.87

Statistical analysis was performed using Fisher’s exact test.

Table 4 summarizes the clinical characteristics of patients in whom abnormal pulmonary lesions were first detected on HRCT. In this subgroup analysis there were no significant differences between HTLV-1-positive and HTLV-1-negative patients in the seroprevalence of ACPA or RF. Although both groups were within the LDA range for RA, disease activity scores tended to be higher in HTLV-1-positive patients than in HTLV-1-negative patients; however, these differences did not reach statistical significance (DAS28-ESR: 3.39 *vs* 2.54, *P* = 0.05; CDAI: 5.90 *vs* 3.05, *P* = 0.08; SDAI: 6.37 *vs* 3.28, *P* = 0.06). The proportion of patients treated with glucocorticoids was also higher in the HTLV-1-positive group than in the HTLV-1-negative group (86% *vs* 50%, *P* = 0.03), while fewer patients were treated with biologic agents in the HTLV-1-positive group compared with the HTLV-1-negative group (14% *vs* 64%; *P < *0.01). Lastly, there were no differences between the two groups regarding the prevalence of comorbidities, such as CTD, diabetes, skin disease, uveitis and malignancies.

**Table 4:** Baseline characteristics of HTLV-1-negative and positive RA with abnormal pulmonary lesions.

Characteristics	HTLV-1-negative RA (*n* = 48)	HTLV-1-positive RA (*n* = 14)	*P*-value^f^
Female, *n* (%)	38 (79)	11 (78)	>0.99
Age, mean (s.d.), years	76.20 (6.96)	71.00 (7.24)	0.05
Age at diagnosis, mean (s.d.), years	57.60 (13.30)	57.00 (11.11)	0.87
RA duration, median (IQR), years	17.82 (9.00–23.25)	13.50 (8.25–16.50)	0.36
Late-onset RA, *n* (%)	15 (31)	4 (29)	>0.99
Smoker, *n* (%)	11/45 (24)	3 (21)	>0.99
Positive for RF, *n* (%)^a^	35 (73)	11 (79)	>0.99
Positive for ACPA, *n* (%)^b^	38/47 (81)	12 (86)	>0.99
ACPA, median (IQR), U/ml	85.1^d^ (14.25–101.00)	34.3 (28.4–101.00)	0.73
RA stage, *n*	I :0; II: 10; III: 14; IV: 24	I: 1; II: 3; III: 3; IV: 7	0.30
RA class, *n*	I: 29; II: 10; III: 5; IV: 4	I: 7; II: 5; III: 2; IV: 0	0.47
RA disease activity			
MMP-3, median (IQR)	91.7 (57.80–142.30)	108.2^c^ (57.25–265.15)	0.49
CRP, median (IQR)	0.13 (0.04–0.60)	0.30 (0.13–0.94)	0.11
DAS28-ESR, mean (SD)	2.54^d^ (1.16)	3.39 (1.03)	0.05
CDAI, median (IQR)	3.05^d^ (0.88–5.88)	5.90 (3.73–9.05)	0.08
SDAI, median (IQR)	3.28^d^ (1.10–6.30)	6.37 (4.01–10.99)	0.06
Pain, 100-mm VAS, median (IQR)	11.5^e^ (3.00–30.00)	33.5 (6.25–49.5)	0.13
PGA, 100-mm VAS, median (IQR)	13^d^ (4.00–30.75)	33.5 (8.75–56.75)	0.09
EGA, 100-mm VAS, median (IQR)	5^d^ (3.00–12.00)	10 (3.50–14.25)	0.09
HAQ-DI, median (IQR)	0.375^d^ (0.00–1.55)	0.75 (0.00–1.40)	0.96
Treatment, *n* (%)			
Corticosteroid	24 (50)	12 (86)	0.03
Methotrexate	19 (40)	5 (35)	>0.99
Biologics	31 (64)	2 (14)	<0.01
Tacrolimus	14 (29)	5 (35)	0.74
Comorbidities, *n* (%)			
CTD	12 (25)	3 (21)	>0.99
Interstitial pneumonia	26 (54)	10 (71)	0.36
Uveitis	2 (4)	1 (7)	0.54
Skin disease	0	0	>0.99
HAM	–	1 (7)	
ATL	–	1 (7)	
Diabetes	8 (17)	3 (21)	0.70
Malignancy	8 (17)	2 (14)	>0.99

a RF positivity (>15 IU/ml).

b ACPA positivity (≥4.5 U/ml).

c *n* = 11.

d *n* = 47.

e *n* = 46.

f Significance level of the Fisher’s exact test for categorical variables and the Mann–Whitney U test for continuous variables.

### There was no difference in HTLV-1 PVL values regardless of the presence of abnormal findings on pulmonary HRCT

**Figure 1:**
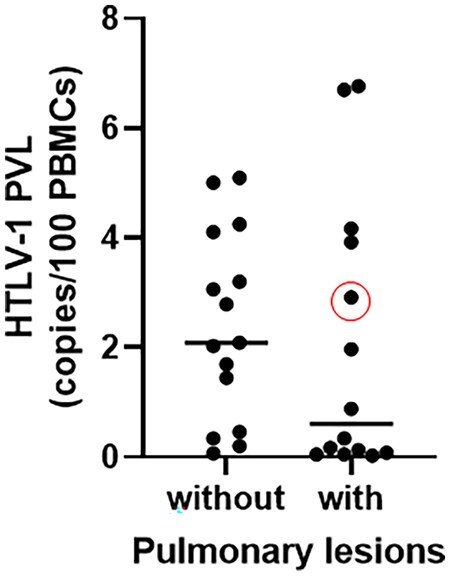
Comparison of the HTLV-1 PVL between patients with and without pulmonary lesions as detected by HRCT. The red circle indicates the PVL of a patient diagnosed with HAM. Statistical analysis was performed using the Mann–Whitney U test. No statistically significant differences were found (0.6 *vs* 2.09 copies/100 PBMCs, *P* = 0.24)

**Figure 2:**
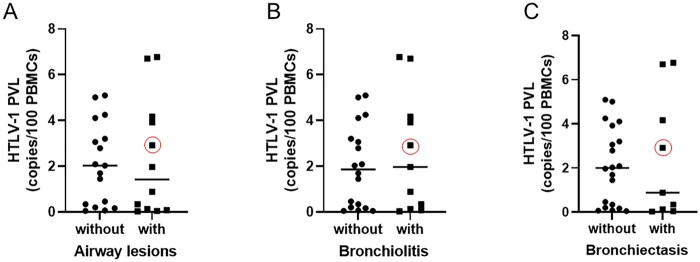
Comparison of the HTLV-1 PVL between patients with and without airway lesions as detected by HRCT. Statistical analysis was performed using the Mann–Whitney U test. **(A)** PVL comparison between patients with and without airway lesions (1.42 *vs* 2.09 copies/100 PBMCs, *P* = 0.52). **(B)** PVL comparison in HTLV-1-positive patients with RA with and without bronchiectasis (1.97 *vs* 2.18 copies/100 PBMCs, *P* = 0.81). **(C)** PVL comparison in HTLV-1-positive patients with RA with and without bronchiolitis (0.88 *vs* 2.00 copies/100 PBMCs, *P* = 0.82). The red circle indicates the PVL of a patient diagnosed with HAM

We compared the HTLV-1 PVL values between the HTLV-1-positive group patients with and without abnormal pulmonary HRCT findings and found no statistically significant differences (0.6 *vs* 2.09 copies/100 PBMCs, *P* = 0.24; Fig. 1). Likewise, there were no statistically significant differences in the HTLV-1 PVL value of HTLV-1-positive patients with and without airway lesions, such as bronchiolitis and bronchiectasis (1.42 *vs* 2.09 copies/100 PBMCs, *P* = 0.52; Fig. 2A) or the presence or absence of bronchiectasis or bronchiolitis (1.97 *vs* 2.18 copies/100 PBMCs, *P* = 0.81 and 0.88 *vs* 2.00 copies/100 PBMCs, *P* = 0.82, respectively; Fig. 2B and C).

## Discussion

Our analysis revealed that the prevalence of pulmonary complications, such as bronchiolitis, in HTLV-1-positive patients with RA is higher than that in HTLV-1-negative patients, suggesting the role of HTLV-1 infection as a possible risk factor for developing pulmonary disorders in patients with RA.

Previous studies have suggested that HTLV-1 infection may alter the clinical features of RA patients [21–23]. HTLV-1-positive patients with RA tend to have severe RA disease activity, especially in terms of pain and general health assessment [24]. In the present study, both pain-VAS and PtGA were higher in the HTLV-1-positive group compared with the HTLV-1-negative group, consistent with previous findings. Furthermore, HTLV-1 infection may contribute to the poorer health assessment of HTLV-1 carriers [25–27]. An epidemiologic systematic review and meta-analysis indicated an association between HTLV-1 infection and rheumatologic disorders, such as fibromyalgia and arthropathy, in HTLV-1 carriers [6]. Although the pathogenesis of the pain associated with HTLV-1 infection has not been elucidated, presumably HTLV-1 infection causes an overall worsening of the patient’s general health assessment related to their pain. The high use of glucocorticoids in HTLV-1-positive patients with RA was likely related to treatment selection rather than a direct cause of increased disease activity. Moreover, the lower use of MTX and bDMARDs, which are anchor drugs for RA, in this group may be attributable to concerns regarding pre-existing pulmonary abnormalities detected on HRCT. Consequently, achieving adequate disease control with MTX and bDMARDs may have been challenging, causing greater reliance on glucocorticoids.

The respiratory system is a common site of extra-articular involvement in RA, with most lung compartments being susceptible to the disease. ILD and airway diseases are the most frequent pulmonary manifestations of RA [28], with airway diseases being particularly prevalent (39–60%) [28] in RA, and any part of the airway may be involved, including the large airways (upper and lower) and distal small airways, and the most common airway lesions include bronchiectasis, bronchiolitis, airway hyperreactivity and cricoarytenoid arthritis [28]. Chest HRCT is useful in diagnosing airway involvement in patients with RA, especially in the respiratory asymptomatic state. In these patients, HRCT imaging is preferred over plane chest radiograph for its superior sensitivity in detecting early pulmonary parenchymal and small airway lesions [29, 30]. In the present study, chest HRCT revealed several pulmonary lesions in both HTLV-1-positive and HTLV-1-negative patients with RA. Notably, the prevalence of bronchiolitis was significantly higher in HTLV-1-positive patients than in HTLV-1-negative patients, whereas bronchiectasis and the coexistence of bronchiolitis and bronchiectasis were more frequently observed in the HTLV-1-positive group, although these differences did not reach statistical significance. Recent studies have suggested that HTLV-1 infection may be involved in pulmonary injury in HTLV-1 carriers. HABA is a well-recognized HTLV-1-related respiratory disorder [8], typically characterized by pulmonary findings such as bronchiectasis, bronchiolitis or a combination of both [9]. Bronchiectasis has also been reported as a common pulmonary manifestation in HTLV-1 carriers and in patients with HAM/TSP [31, 32]. Increased numbers of activated CD4^+^, CD8^+^, CD25^+^ and CD4^+^CD29^+^ T cells have been identified in the bronchoalveolar lavage fluid (BALF) of patients with HTLV-1-associated pulmonary disease, particularly those with HAM/TSP [33]. Similarly, higher proportions of CD3^+^CD25^+^ T cells have been observed in HTLV-1-infected patients with diffuse panbronchiolitis and cryptogenic fibrosing alveolitis compared with uninfected patients [34]. Although the precise pathogenesis of respiratory lesions in HTLV-1 carriers remains unclear, these activated T cells, including HTLV-1-infected T cells, may contribute to lung injury [7]. Therefore, HTLV-1 infection may play a role in the development and progression of airway-dominant pulmonary lesions, particularly bronchiolitis, in patients with RA.

It is well-known that the main target of HTLV-1 infection is CD4^+^ lymphocytes. Several studies have indicated that the function of HTLV-1-infected CD4^+^ lymphocytes is altered similar to a type 1 helper T cell, and the accumulation of HTLV-1-infected cells in the lungs creates an inflammatory milieu [35]. Several studies have described elevated levels of cytokines, including IFN-γ and IL-2 [36, 37], and chemokines such as CCL3, CCL5 and CXCL10 [37, 38] in the BALF of patients with HTLV-1 infection compared with uninfected controls. The local production of inflammatory cytokines and chemokines may be associated with the infiltration of transcriptionally active HTLV-1-infected lymphocytes into the lungs. Therefore, apart from HTLV-1-associated inflammatory disorders, such as HAM/TSP, HTLV-1-infected cells may be involved in the pathogenesis of respiratory manifestations of RA [8].

Patients with HAM/TSP have been reported to exhibit abnormal chest CT findings more frequently than asymptomatic HTLV-1 carriers [39], and HTLV-1-related pulmonary disease may be progressive, with the development of new lung lesions, particularly in patients with HAM/TSP [40]. Because patients with HAM/TSP generally have higher HTLV-1 PVL than asymptomatic carriers, elevated PVL has been considered a potential risk factor for pulmonary involvement. However, in the present study, no significant differences in HTLV-1 PVL were observed according to the presence or absence of airway lesions, including bronchiolitis and bronchiectasis, among HTLV-1-positive patients with RA. Similarly, a cohort study from the UK reported no independent association between bronchiectasis and high HTLV-1 PVL in multivariate analyses [40]. Instead, the mentioned study suggests that comorbid inflammatory conditions, such as HAM/TSP, myositis, HAU and inflammatory arthritis, were more strongly associated with the prevalence of bronchiectasis in HTLV-1 carriers than PVL itself [32]. RA is a systemic inflammatory disease that may involve extra-articular manifestations, including pulmonary involvement. In patients with RA who are predisposed to lung lesions, infiltration of HTLV-1-infected or activated T cells into pulmonary tissue may occur more readily, potentially causing airway-dominant lung damage irrespective of HTLV-1 PVL. This hypothesis is supported by the absence of a clear association between PVL and airway lesions in our cohort and suggests that the host’s inflammatory status may play a more prominent role than the viral burden alone. Further studies incorporating bronchoalveolar lavage fluid analyses are required to clarify whether an increased number of HTLV-1-infected cells or activated T cells contribute to pulmonary involvement in HTLV-1-positive patients with RA. Previous studies from Australia have reported a strong association between high HTLV-1 PVL and bronchiectasis, particularly in populations infected with HTLV-1 subtype C (Australo-Melanesian), which is unique to central Australia [36, 41, 42]. In contrast, such a strong association was not observed in the present study or in the UK cohort. Several factors may account for these discrepancies, including the relatively small number of HTLV-1-positive patients in our cohort; differences in genetic background, viral subtypes and environmental exposures; and variations in study design and selection criteria, such as indications for HRCT and definitions of bronchiectasis. Therefore, the absence of a significant association between HTLV-1 PVL and airway lesions in the current study should be interpreted with caution. Importantly, the pulmonary abnormalities identified in this study are best regarded as representing an airway-predominant pattern of involvement rather than a discrete pathological entity, because airway lesions such as bronchiolitis, bronchiolectasis and non-traction bronchiectasis may overlap with other forms of pulmonary involvement.

Patients with RA have a higher risk of developing infections compared with non-RA subjects [43], with particularly higher chances of respiratory infections in patients with RA who have comorbid pulmonary lesions [44, 45]. Therefore, an increased prevalence of pulmonary lesions due to HTLV-1 infection may increase the risk of developing respiratory infections in HTLV-1-positive patients with RA. A previous study by Hashiba *et al.* [46] suggested that HTLV-1-positive patients with RA had a high incidence of serious infection compared with HTLV-1-negative patients with RA. The worsening of respiratory infections is frequently caused by the hospitalization of HTLV-1-positive patients with RA, indicating that a high prevalence of airway lesions in HTLV-1-positive patients with RA may be associated with the need for hospitalization for worsening of respiratory infections. Future studies should aim to elucidate whether HTLV-1 infection is a potential risk factor for developing respiratory complications and respiratory infections in HTLV-1-positive patients with RA.

This study has several limitations. First, this study included a relatively small number of HTLV-1-positive patients, which limits statistical power and increases the possibility of chance findings in some observed associations. In particular, the absence of a significant association between HTLV-1 PVL and pulmonary or airway lesions should be interpreted with caution, as this finding may reflect limited sample size or assay sensitivity rather than a true lack of biological relevance. Accordingly, the present results should be regarded as hypothesis generating rather than definitive. Second, HRCT imaging was not performed in all patients enrolled in the registry but was instead conducted based on clinical or radiological indications suggestive of pulmonary involvement at the initiation of antirheumatic therapy. Given that HRCT was mainly performed for clinical indications rather than for asymptomatic screening, the absolute prevalence of pulmonary abnormalities may have been overestimated. However, considering the similar indications for HRCT between HTLV-1-positive and -negative patients, comparative analyses between the groups would have unlikely been substantially biased. Third, interobserver variability in HRCT interpretation was not formally evaluated. It is crucial to acknowledge that interobserver agreement for CT-based classification of ILD patterns, including the UIP pattern, is often only moderate [47]. Furthermore, identifying subtle airway findings such as non-traction bronchiectasis and bronchiolectasis may be even more challenging and could have influenced lesion classification in the present study. Although we attempted to minimize this bias through independent review and a consensus-based process, formal agreement analysis (e.g. κ statistics) was not performed. Therefore, our results should be interpreted considering these radiological limitations. Fourth, this study failed to clarify whether the pulmonary lesions observed in HTLV-1-positive patients with RA shared the same pathological features as HTLV-1-associated pulmonary disorders, such as HABA. Further studies incorporating BALF analyses, including assessment of HTLV-1-infected cell proportions and cytokine or chemokine profiles, are needed to elucidate the mechanisms underlying lung involvement in this population. Finally, given that this was a single-region study conducted in an HTLV-1-endemic area in Japan, our findings may not be generalized to non-endemic regions or populations with different genetic or environmental backgrounds. Moreover, this study focused specifically on HTLV-1 due to its unique biological characteristics, including lifelong persistence and T cell tropism. However, the potential influence of viral infections other than HTLV-1 on pulmonary manifestations in patients with RA was not evaluated and therefore cannot be excluded.

In summary, this study demonstrated that airway-dominant pulmonary lesions, particularly bronchiolitis, were more frequently observed in HTLV-1-positive patients with RA than in HTLV-1-negative patients, despite comparable overall rates of pulmonary abnormalities. Although no significant differences in HTLV-1 PVL were identified according to the presence or absence of lung lesions, the higher prevalence of airway involvement suggests a potential association between HTLV-1 infection and respiratory comorbidities in RA. From a clinical perspective, these findings suggest that, in HTLV-1-endemic regions, assessment of HTLV-1 infection status may be clinically informative in selected patients with RA who present with airway-dominant pulmonary lesions or recurrent respiratory infections, as it may help guide pulmonary evaluation and antirheumatic treatment decision-making. However, routine screening of all patients with RA cannot be recommended based on the present data.

## Supplementary Material

rkag046_Supplementary_Data

## Data Availability

The data underlying this article will be shared upon reasonable request to the corresponding author.

## References

[1] Smolen JS , Aletaha D, McInnes IB. Rheumatoid arthritis. Lancet 2016;388:2023–38. 27156434 10.1016/S0140-6736(16)30173-8

[2] Shaw M , Collins BF, Ho LA, Raghu G. Rheumatoid arthritis-associated lung disease. Eur Respir Rev 2015;24:1–16. 25726549 10.1183/09059180.00008014 PMC9487778

[3] Nakajima A , Inoue E, Tanaka E et al Mortality and cause of death in Japanese patients with rheumatoid arthritis based on a large observational cohort, IORRA. Scand J Rheumatol 2010;39:360–7. 20476859 10.3109/03009741003604542

[4] Antoniou KM , Distler O, Gheorghiu A-M et al ERS/EULAR clinical practice guidelines for connective tissue disease-associated interstitial lung disease developed by the task force for connective tissue disease-associated interstitial lung disease of the European Respiratory Society (ERS) and the European Alliance of Associations for Rheumatology (EULAR) Endorsed by the European Reference Network on rare respiratory diseases (ERN-LUNG). Ann Rheum Dis 2026;85:22–60. 40912974 10.1016/j.ard.2025.08.021

[5] Iwanaga M , Watanabe T, Yamaguchi K. Adult T-cell leukemia: a review of epidemiological evidence. Front Microbiol 2012;3:322. 22973265 10.3389/fmicb.2012.00322 PMC3437524

[6] Schierhout G , McGregor S, Gessain A et al Association between HTLV-1 infection and adverse health outcomes: a systematic review and meta-analysis of epidemiological studies. Lancet Infect Dis 2020;20:133–43. 31648940 10.1016/S1473-3099(19)30402-5

[7] Gallo RC , Tagaya Y. Reflections on some of the exceptional features of HTLV-1 and HTLV-1 research: a perspective. Front Immunol 2022;13:859654. 35432297 10.3389/fimmu.2022.859654 PMC9010860

[8] Dias ÁRN , Falcão LFM, Quaresma JAS. An overview of human T-lymphotropic virus type 1 lung injury. Front Immunol 2022;13:914498. 35844492 10.3389/fimmu.2022.914498 PMC9285117

[9] Arnett FC , Edworthy SM, Bloch DA et al The American Rheumatism Association 1987 revised criteria for the classification of rheumatoid arthritis. Arthritis Rheum 1988;31:315–24. 3358796 10.1002/art.1780310302

[10] Aletaha D , Neogi T, Silman AJ et al 2010 rheumatoid arthritis classification criteria: an American College of Rheumatology/European League Against Rheumatism collaborative initiative. Arthritis Rheum 2010;62:2569–81. 20872595 10.1002/art.27584

[11] Singh JA , Saag KG, Bridges SL et al 2015 American College of Rheumatology guideline for the treatment of rheumatoid arthritis. Arthritis Care Res (Hoboken) 2016;68:1–25. 26545825 10.1002/acr.22783

[12] Smolen JS , Landewé R, Bijlsma J et al EULAR recommendations for the management of rheumatoid arthritis with synthetic and biological disease-modifying antirheumatic drugs: 2016 update. Ann Rheum Dis 2017;76:960–77. 28264816 10.1136/annrheumdis-2016-210715

[13] Kerschbaumer A , Sepriano A, Smolen JS et al Efficacy of pharmacological treatment in rheumatoid arthritis: a systematic literature research informing the 2019 update of the EULAR recommendations for management of rheumatoid arthritis. Ann Rheum Dis 2020;79:744–59. 32033937 10.1136/annrheumdis-2019-216656 PMC7286044

[14] Umekita K , Hashiba Y, Kariya Y et al The time-sequential changes of risk factors for adult T-cell leukemia development in human T-cell leukemia virus-positive patients with rheumatoid arthritis: a retrospective cohort study. Mod Rheumatol 2019;29:795–801. 30246572 10.1080/14397595.2018.1519890

[15] Eba J , Nakamura K. Overview of the ethical guidelines for medical and biological research involving human subjects in Japan. Jpn J Clin Oncol 2022;52:539–44. 35349681 10.1093/jjco/hyac034 PMC9157286

[16] van Gestel AM , Haagsma CJ, van Riel PL. Validation of rheumatoid arthritis improvement criteria that include simplified joint counts. Arthritis Rheum 1998;41:1845–50. 9778226 10.1002/1529-0131(199810)41:10<1845::AID-ART17>3.0.CO;2-K

[17] Anderson JK , Zimmerman L, Caplan L, Michaud K. Measures of rheumatoid arthritis disease activity: Patient (PtGA) and Provider (PrGA) Global Assessment of Disease Activity, Disease Activity Score (DAS) and Disease Activity Score with 28-joint counts (DAS28), Simplified Disease Activity Index (SDAI), Clinical Disease Activity Index (CDAI), Patient Activity Score (PAS) and Patient Activity Score-II (PASII), Routine Assessment of Patient Index Data (RAPID), Rheumatoid Arthritis Disease Activity Index (RADAI) and Rheumatoid Arthritis Disease Activity Index-5 (RADAI-5), Chronic Arthritis Systemic Index (CASI), Patient-based Disease Activity Score with ESR (PDAS1) and Patient-based Disease Activity Score without ESR (PDAS2), and Mean Overall Index for Rheumatoid Arthritis (MOI-RA). Arthritis Care Res (Hoboken). 2011;63(Suppl 11):S14–36. 22588741 10.1002/acr.20621

[18] Prevoo ML , van ’t Hof MA, Kuper HH et al Modified disease activity scores that include twenty-eight-joint counts. Development and validation in a prospective longitudinal study of patients with rheumatoid arthritis. Arthritis Rheum 1995;38:44–8. 7818570 10.1002/art.1780380107

[19] Felson DT , Smolen JS, Wells G et al American College of Rheumatology/European League Against Rheumatism provisional definition of remission in rheumatoid arthritis for clinical trials. Arthritis Rheum 2011;63:573–86. 21294106 10.1002/art.30129 PMC3115717

[20] Tanaka G-I , Okayama A, Watanabe T et al The clonal expansion of human T lymphotropic virus type 1-infected T cells: a comparison between seroconverters and long-term carriers. J Infect Dis 2005;191:1140–7. 15747250 10.1086/428625

[21] Umekita K , Hidaka T, Miyauchi S et al Treatment with anti–tumor necrosis factor biologic agents in human T lymphotropic virus type I–positive patients with rheumatoid arthritis. Arthritis Care Res 2014;66:788–92. 10.1002/acr.22205 24127184

[22] Umekita K , Hashiba Y, Iwao K et al Human T-cell leukemia virus type 1 may invalidate T-SPOT.TB assay results in rheumatoid arthritis patients: a retrospective case-control observational study. PLoS One 2020;15:e0233159. 32459801 10.1371/journal.pone.0233159 PMC7252607

[23] Umekita K. Effect of HTLV-1 infection on the clinical course of patients with rheumatoid arthritis. Viruses 2022;14:1460. 35891440 10.3390/v14071460 PMC9323945

[24] Endo Y , Fukui S, Umekita K et al Effectiveness and safety of non-tumor necrosis factor inhibitor therapy for anti-human T-cell leukemia virus type 1 antibody-positive rheumatoid arthritis. Mod Rheumatol 2021;31:972–8. 33161771 10.1080/14397595.2020.1847802

[25] San-Martin DL , Santos DN, Baptista AF, Pain Study Group. Pain prevalence, characteristics and associated factors in human T-cell lymphotropic virus type 1 infected patients: a systematic review of the literature. Braz J Infect Dis 2016;20:592–8. 27768899 10.1016/j.bjid.2016.08.013 PMC9427562

[26] Poetker SKW , Porto AF, Giozza SP et al Clinical manifestations in individuals with recent diagnosis of HTLV type I infection. J Clin Virol 2011;51:54–8. 21388871 10.1016/j.jcv.2011.02.004 PMC3074002

[27] Santos DND , Santos KOB, Paixão AB et al Factors associated with pain in individuals infected by human T-cell lymphotropic virus type 1 (HTLV-1). Braz J Infect Dis 2017;21:133–9. 28011062 10.1016/j.bjid.2016.11.008 PMC9427659

[28] Yunt ZX , Solomon JJ. Lung disease in rheumatoid arthritis. Rheum Dis Clin North Am 2015;41:225–36. 25836639 10.1016/j.rdc.2014.12.004 PMC4415514

[29] Johnson SR , Bernstein EJ, Bolster MB et al 2023 American College of Rheumatology (ACR)/American College of Chest Physicians (CHEST) guideline for the screening and monitoring of interstitial lung disease in people with systemic autoimmune rheumatic diseases. Arthritis Rheumatol 2024;76:1201–13. 38973714 10.1002/art.42860 PMC12646464

[30] Guiot J , Miedema J, Cordeiro A et al Practical guidance for the early recognition and follow-up of patients with connective tissue disease-related interstitial lung disease. Autoimmun Rev 2024;23:103582. 39074630 10.1016/j.autrev.2024.103582

[31] Okada F , Ando Y, Yoshitake S et al Pulmonary CT findings in 320 carriers of human T-lymphotropic virus type 1. Radiology 2006;240:559–64. 16864677 10.1148/radiol.2402050886

[32] Honarbakhsh S , Taylor GP. High prevalence of bronchiectasis is linked to HTLV-1-associated inflammatory disease. BMC Infect Dis 2015;15:258. 26143070 10.1186/s12879-015-1002-0 PMC4491414

[33] Mukae H , Kohno S, Morikawa N et al Increase in T-cells bearing CD25 in bronchoalveolar lavage fluid from HAM/TSP patients and HTLV-I carriers. Microbiol Immunol 1994;38:55–62. 8052162 10.1111/j.1348-0421.1994.tb01744.x

[34] Yamamoto M , Matsuyama W, Oonakahara K et al Influence of human T lymphotrophic virus type I on diffuse pan-bronchiolitis. Clin Exp Immunol 2004;136:513–20. 15147354 10.1111/j.1365-2249.2004.02485.x PMC1809062

[35] Einsiedel L , Chiong F, Jersmann H, Taylor GP. Human T-cell leukaemia virus type 1 associated pulmonary disease: clinical and pathological features of an under-recognised complication of HTLV-1 infection. Retrovirology 2021;18:1. 33407607 10.1186/s12977-020-00543-z PMC7789585

[36] Nakayama Y , Yamazato Y, Tamayose M et al Increased expression of HBZ and Foxp3 mRNA in bronchoalveolar lavage cells taken from human T-lymphotropic virus type 1-associated lung disorder patients. Intern Med 2013;52:2599–609. 24292748 10.2169/internalmedicine.52.0845

[37] Yamazato Y , Miyazato A, Kawakami K et al High expression of p40*tax* and pro-inflammatory cytokines and chemokines in the lungs of human T-lymphotropic virus type 1-related bronchopulmonary disorders. Chest 2003;124:2283–92. 14665512 10.1378/chest.124.6.2283

[38] Seki M , Kadota JI, Higashiyama Y et al Elevated levels of beta-chemokines in bronchoalveolar lavage fluid (BALF) of individuals infected with human T lymphotropic virus type-1 (HTLV-1). Clin Exp Immunol 1999;118:417–22. 10594561 10.1046/j.1365-2249.1999.01093.x PMC1905436

[39] Magno Falcão LF , Falcão ASC, Medeiros Sousa RC et al CT chest and pulmonary functional changes in patients with HTLV-associated myelopathy in the eastern Brazilian Amazon. PLoS One 2017;12:e0186055. 29095831 10.1371/journal.pone.0186055 PMC5667869

[40] Dias ARN , Vieira WdB, Normando VMF et al Computed tomography with 6-year follow-up demonstrates the evolution of HTLV-1 related lung injuries: a cohort study. PLoS One 2021;16:e0261864. 34965281 10.1371/journal.pone.0261864 PMC8716036

[41] Einsiedel L , Fernandes L, Spelman T, Steinfort D, Gotuzzo E. Bronchiectasis is associated with human T-lymphotropic virus 1 infection in an Indigenous Australian population. Clin Infect Dis 2012;54:43–50. 22095566 10.1093/cid/cir766

[42] Einsiedel L , Cassar O, Goeman E et al Higher human T-lymphotropic virus type 1 subtype C proviral loads are associated with bronchiectasis in indigenous Australians: results of a case-control study. Open Forum Infect Dis 2014;1:ofu023. 25734096 10.1093/ofid/ofu023 PMC4324180

[43] Doran MF , Crowson CS, Pond GR, O’Fallon WM, Gabriel SE. Frequency of infection in patients with rheumatoid arthritis compared with controls: a population-based study. Arthritis Rheum 2002;46:2287–93. 12355475 10.1002/art.10524

[44] Coyne P , Hamilton J, Heycock C et al Acute lower respiratory tract infections in patients with rheumatoid arthritis. J Rheumatol 2007;34:1832–6. 17659759

[45] Honne K , Bando M, Mieno MN, Iwamoto M, Minota S. Bronchiectasis is as crucial as interstitial lung disease in the severe pneumonia that occurs during treatment with biologic DMARDs in rheumatoid arthritis: a retrospective cohort study in a single facility. Rheumatol Int 2022;42:1341–6. 34251498 10.1007/s00296-021-04934-z

[46] Hashiba Y , Umekita K, Kimura M et al High incidence of serious infections requiring hospitalisation in human T-cell leukaemia virus type 1-positive rheumatoid arthritis: a case-controlled observational study. Mod Rheumatol 2022;32:866–74. 34897491 10.1093/mr/roab077

[47] Walsh SL , Calandriello L, Sverzellati N, Wells AU, Hansell DM. Interobserver agreement for the ATS/ERS/JRS/ALAT criteria for a UIP pattern on CT. Thorax 2016;71:45–51. 26585524 10.1136/thoraxjnl-2015-207252

