# Supplementary material for: The prevalence of pulmonary airway lesions is high in HTLV-1-positive rheumatoid arthritis: a cross-sectional observational study
Source: Rheumatol Adv Pract. 2026 Apr 7;10(2):rkag046. doi: 10.1093/rap/rkag046 (PMC13134664; doi:10.1093/rap/rkag046)
Supplement: rkag046_Supplementary_Data [file rkag046_supplementary_data.docx]

**Supplementary Table S1. Comparison of clinical characteristics and HRCT findings between HTLV-1-negative and HTLV-1-positive RA patients before and after propensity score matching (matched by age, sex, and b/tsDMARDs use).**

|  | Before Matching | | | After Matching | | |
| --- | --- | --- | --- | --- | --- | --- |
|  | HTLV-1(-)RA  (n = 90) | HTLV-1(+)RA  (n = 30) | P-value | HTLV-1(-)RA  (n = 14) | HTLV-1(+)RA  (n = 14) | P-value |
| Age, mean (SD) | 73.36 (8.55) | 69.57 (9.28) | 0.042 | 72.79 (5.58) | 72.79 (5.58) | >0.99 |
| Sex(female) | 75 (83%) | 24 (80%) | 0.68 | 2 (14%) | 2 (14%) | >0.99 |
| Age at diagnosis, mean (SD) | 54.98 (13.26) | 55.60 (11.55) | 0.82 | 56.07 (12.95) | 56.57 (13.37) | 0.92 |
| RA duration, median (IQR) | 15.50 (9.00, 25.00) | 13.50 (7.00, 20.00) | 0.11 | 12.50 (8.00, 25.00) | 17.00 (7.00, 22.00) | 0.94 |
| ACPA, median (IQR) | 61.30 (7.00, 101.00) | 28.60 (5.40, 101.00) | 0.29 | 77.85 (2.50, 101.00) | 15.80 (4.50, 82.60) | 0.4 |
| MMP-3, median (IQR) | 73.15 (47.20, 134.00) | 105.70 (47.70, 209.50) | 0.27 | 44.95 (37.50, 74.60) | 105.70 (51.80, 209.50) | 0.065 |
| CRP, median (IQR) | 0.11 (0.04, 0.33) | 0.14 (0.05, 0.82) | 0.37 | 0.13 (0.04, 0.60) | 0.17 (0.03, 0.82) | 0.82 |
| DAS28-ESR, mean (SD) | 2.56 (1.05) | 3.00 (1.08) | 0.060 | 2.26 (1.10) | 3.09 (0.95) | 0.054 |
| CDAI, median (IQR) | 3.30 (1.15, 5.85) | 6.40 (3.05, 9.85) | 0.012 | 1.85 (0.40, 5.00) | 6.10 (2.85, 9.00) | 0.05 |
| SDAI, median (IQR) | 3.46 (1.50, 6.38) | 7.28 (3.22, 11.30) | 0.011 | 1.94 (0.90, 6.44) | 6.77 (2.88, 10.80) | 0.08 |
| Pain, 100 mm VAS, median (IQR) | 12.00 (4.00, 30.00) | 27.00 (10.00, 51.00) | 0.023 | 8.00 (1.00, 15.00) | 25.00 (11.00, 35.00) | 0.027 |
| PGA, 100 mm VAS, median (IQR) | 14.00 (5.00, 31.00) | 30.00 (13.00, 53.00) | 0.031 | 8.50 (2.00, 15.00) | 28.00 (16.00, 37.00) | 0.018 |
| EGA, 100 mm VAS, median (IQR) | 5.00 (3.00, 11.00) | 10.00 (3.00, 15.00) | 0.045 | 3.00 (3.00, 7.00) | 10.00 (6.00, 12.00) | 0.03 |
| HAQ_DI, median (IQR) | 0.38 (0.00, 1.40) | 0.88 (0.12, 1.50) | 0.28 | 0.35 (0.00, 1.50) | 1.30 (0.12, 1.50) | 0.41 |
| Abnormal Findings(total) | 48 (53%) | 14 (47%) | 0.53 | 6 (43%) | 5 (36%) | 0.7 |
| Bronchiolitis | 12 (13%) | 11 (37%) | 0.005 | 12 (86%) | 10 (71%) | 0.36 |
| Bronchiecrtasis | 15 (17%) | 9 (30%) | 0.11 | 4 (29%) | 4 (29%) | 1.00 |
| Reticular pattern | 21 (23%) | 7 (23%) | >0.99 | 2 (14%) | 2 (14%) | 1.00 |
| Interlobular septum thickening | 19 (21%) | 7 (23%) | 0.80 | 2 (14%) | 1 (7%) | 0.54 |
| Ground glass attenuation | 8 (9%) | 4 (13%) | 0.48 | 1 (7%) | 1 (7%) | >0.99 |
| Honeycoming | 11 (12%) | 4 (13%) | 0.87 | 1 (7%) | 0 (0%) | 0.31 |
| Pleural thickening | 11 (12%) | 3 (10%) | 0.74 | 1 (7%) | 0 (0%) | 0.31 |
| Lung nodule | 5 (6%) | 2 (7%) | 0.82 | 0 (0%) | 0 (0%) | >0.99 |
| Emphysematous bullae | 4 (4%) | 1 (3%) | 0.79 | 0 (0%) | 0 (0%) | >0.99 |
| ILD | 26 (29%) | 10 (33%) | 0.65 | 13 (93%) | 14 (100%) | 0.36 |
| UIP | 11 (12%) | 4 (13%) | 0.87 | 12 (86%) | 14 (100%) | 0.31 |
| NSIP | 4 (4%) | 1 (3%) | 0.79 | 2 (14%) | 0 (0%) | 0.14 |
| OP | 0 (0%) | 1 (3%) | 0.082 | 0 (0%) | 0 (0%) | >0.99 |
| Mild, Unclassifiable | 11 (12%) | 4 (13%) | 0.87 | 1 (7%) | 2 (14%) | 0.54 |
| Methotrexate | 47 (52%) | 10 (33%) | 0.073 | 9 (64%) | 3 (21%) | 0.022 |
| corticosteroid | 32 (36%) | 21 (70%) | 0.001 | 3 (21%) | 9 (64%) | 0.022 |
| bDMARDs | 55 (61%) | 10 (33%) | 0.008 | 5 (36%) | 5 (36%) | >0.99 |

Data are presented as mean (SD), n (%), or median (IQR). HTLV-1: human T-cell leukemia virus type 1; RA: rheumatoid arthritis; SMD: standardized mean difference; SD: standard deviation; IQR: interquartile range; ACPA: anti-citrullinated protein antibody; RF: rheumatoid factor; DAS28-ESR: disease activity score in 28 joints with erythrocyte sedimentation rate; SDAI: simplified disease activity index; CDAI: clinical disease activity index; VAS: visual analogue scale; PGA, patient global assessment; EGA, evaluator global assessment; HAQ_DI: Health Assessment Questionnaire Disability Index; ILD: interstitial lung disease; UIP: usual interstitial pneumonia; NSIP: non-specific interstitial pneumonia; OP: organizing pneumonia; bDMARDs: biological antirheumatic drugs. *Abnormal Findings (total) include bronchiolitis, bronchiectasis, reticular pattern, interlobular septal thickening, ground-glass attenuation, honeycombing, pleural thickening, lung nodules, and emphysematous bullae, and interstitial pneumonia.

**Supplementary Table S2. Comparison of clinical characteristics and HRCT findings before and after propensity score matching (matched by disease activity [DAS28-ESR, CDAI, SDAI]).**

|  | Before Matching | | | After Matching | | |
| --- | --- | --- | --- | --- | --- | --- |
|  | HTLV-1(-)RA  (n = 90) | HTLV-1(+)RA  (n = 30) | P-value | HTLV-1(-)RA  (n = 9) | HTLV-1(+)RA  (n = 9) | P-value |
| Age, mean (SD) | 73.36 (8.55) | 69.57 (9.28) | 0.042 | 72.33 (6.54) | 68.56 (8.31) | 0.30 |
| Sex | 75 (83%) | 24 (80%) | 0.68 | 7 (78%) | 7 (78%) | >0.99 |
| Age at diagnosis, mean (SD) | 54.98 (13.26) | 55.60 (11.55) | 0.82 | 61.67 (8.93) | 56.22 (10.10) | 0.24 |
| RA duration, median (IQR) | 15.50 (9.00, 25.00) | 13.50 (7.00, 20.00) | 0.11 | 9.00 (7.00, 13.00) | 12.00 (7.00, 14.00) | 0.69 |
| ACPA, median (IQR) | 61.30 (7.00, 101.00) | 28.60 (5.40, 101.00) | 0.29 | 101.00 (7.50, 101.00) | 82.60 (21.70, 101.00) | 0.74 |
| MMP-3, median (IQR) | 73.15 (47.20, 134.00) | 105.70 (47.70, 209.50) | 0.27 | 71.70 (58.80, 89.60) | 51.80 (41.30, 166.80) | 0.35 |
| CRP, median (IQR) | 0.11 (0.04, 0.33) | 0.14 (0.05, 0.82) | 0.37 | 0.09 (0.06, 0.52) | 0.14 (0.03, 0.23) | 0.63 |
| DAS28-ESR, mean (SD) | 2.56 (1.05) | 3.00 (1.08) | 0.060 | 2.22 (1.03) | 2.31 (0.78) | 0.84 |
| CDAI, median (IQR) | 3.30 (1.15, 5.85) | 6.40 (3.05, 9.85) | 0.012 | 1.70 (0.60, 4.80) | 3.30 (1.20, 5.00) | 0.69 |
| SDAI, median (IQR) | 3.46 (1.50, 6.38) | 7.28 (3.22, 11.30) | 0.011 | 2.25 (0.90, 5.32) | 3.50 (1.34, 5.52) | 0.76 |
| Pain, 100 mm VAS, median (IQR) | 12.00 (4.00, 30.00) | 27.00 (10.00, 51.00) | 0.023 | 6.00 (1.00, 18.00) | 11.00 (4.00, 31.00) | 0.35 |
| PGA, 100 mm VAS, median (IQR) | 14.00 (5.00, 31.00) | 30.00 (13.00, 53.00) | 0.031 | 5.00 (1.00, 19.00) | 16.00 (3.00, 30.00) | 0.45 |
| EGA, 100 mm VAS, median (IQR) | 5.00 (3.00, 11.00) | 10.00 (3.00, 15.00) | 0.045 | 5.00 (3.00, 15.00) | 3.00 (3.00, 10.00) | 0.49 |
| HAQ_DI, median (IQR) | 0.38 (0.00, 1.40) | 0.88 (0.12, 1.50) | 0.28 | 0.00 (0.00, 0.10) | 0.40 (0.00, 1.00) | 0.25 |
| Abnormal Findings(total) | 48 (53%) | 14 (47%) | 0.53 | 5 (56%) | 5 (56%) | >0.99 |
| Bronchiolitis | 12 (13%) | 11 (37%) | 0.005 | 0 (0%) | 4 (44%) | 0.023 |
| Bronchiecrtasis | 15 (17%) | 9 (30%) | 0.11 | 1 (11%) | 2 (22%) | 0.53 |
| Reticular pattern | 21 (23%) | 7 (23%) | >0.99 | 4 (44%) | 2 (22%) | 0.32 |
| Interlobular septum thickening | 19 (21%) | 7 (23%) | 0.80 | 3 (33%) | 4 (44%) | 0.63 |
| Ground glass attenuation | 8 (9%) | 4 (13%) | 0.48 | 1 (11%) | 1 (11%) | >0.99 |
| Honeycoming | 11 (12%) | 4 (13%) | 0.87 | 1 (11%) | 2 (22%) | 0.53 |
| Pleural thickening | 11 (12%) | 3 (10%) | 0.74 | 1 (11%) | 1 (11%) | >0.99 |
| Lung nodule | 85 (94%) | 28 (93%) | 0.82 | 0 (0%) | 0 (0%) | >0.99 |
| Emphysematous bullae | 4 (4%) | 1 (3%) | 0.79 | 0 (0%) | 1 (11%) | 0.30 |
| ILD | 26 (29%) | 10 (33%) | 0.65 | 3 (33%) | 4 (44%) | 0.63 |
| UIP | 11 (12%) | 4 (13%) | 0.87 | 1 (11%) | 2 (22%) | 0.53 |
| NSIP | 4 (4%) | 1 (3%) | 0.79 | 1 (11%) | 0 (0%) | 0.30 |
| OP | 0 (0%) | 1 (3%) | 0.082 | 0 (0%) | 0 (0%) | >0.99 |
| Mild, Unclassifiable | 11 (12%) | 4 (13%) | 0.87 | 1 (11%) | 2 (22%) | 0.53 |
| Methotrexate | 47 (52%) | 10 (33%) | 0.073 | 6 (67%) | 4 (44%) | 0.34 |
| Corthcosteroid | 32 (36%) | 21 (70%) | 0.001 | 4 (44%) | 5 (56%) | 0.64 |
| bDMARDs | 55 (61%) | 10 (33%) | 0.008 | 5 (56%) | 5 (56%) | >0.99 |

Data are presented as mean (SD), n (%), or median (IQR). HTLV-1: human T-cell leukemia virus type 1; RA: rheumatoid arthritis; SMD: standardized mean difference; SD: standard deviation; IQR: interquartile range; ACPA: anti-citrullinated protein antibody; RF: rheumatoid factor; DAS28-ESR: disease activity score in 28 joints with erythrocyte sedimentation rate; SDAI: simplified disease activity index; CDAI: clinical disease activity index; VAS: visual analogue scale; PGA, patient global assessment; EGA, evaluator global assessment; HAQ_DI: Health Assessment Questionnaire Disability Index; ILD: interstitial lung disease; UIP: usual interstitial pneumonia; NSIP: non-specific interstitial pneumonia; OP: organizing pneumonia; bDMARDs: biological antirheumatic drugs. *Abnormal Findings (total) include bronchiolitis, bronchiectasis, reticular pattern, interlobular septal thickening, ground-glass attenuation, honeycombing, pleural thickening, lung nodules, and emphysematous bullae, and interstitial pneumonia.
